# Perceived Family Functioning Profile in Adolescents at Clinical High Risk for Psychosis: Rigidity as a Possible Preventive Target

**DOI:** 10.3389/fpsyt.2022.861201

**Published:** 2022-04-15

**Authors:** Melanie Iorio, Erica Casini, Stefano Damiani, Paolo Fusar-Poli, Renato Borgatti, Martina Maria Mensi, Clinical High Risk State for Psychosis Research Group

**Affiliations:** ^1^Department of Brain and Behavioral Sciences, University of Pavia, Pavia, Italy; ^2^Child Neurology and Psychiatry Unit, Institute of Hospitalization and Care With Scientific Character (IRCCS) Mondino Foundation, Pavia, Italy; ^3^Early Psychosis: Interventions and Clinical-Detection (EPIC) Lab, Department of Psychosis Studies, Institute of Psychiatry, Psychology and Neuroscience, King’s College London, London, United Kingdom; ^4^OASIS Service, South London and Maudsley NHS Foundation Trust, London, United Kingdom

**Keywords:** family functioning, psychosis, schizophrenia, risk, prevention, adolescence, CHR-P, perceived stress (PS)

## Abstract

The presence of a positive family relationship has been suggested as a protective factor from parental stress and from the development of full-blown psychosis. However, to date, there is limited research on family functioning in adolescents with psychosis and at clinical high risk for psychosis (CHR-P). This study is aimed at comparing family functioning and perceived stress in parents of adolescents with either CHR-P, early onset psychosis (EOP), or other psychiatric disorders (no CHR-P). As a secondary aim, it will correlate family functioning with parental perceived stress in order to find critical targets of intervention. We conducted a Reporting of Studies Conducted Using Observational Routinely-Collected Health Data (RECORD)—compliant, real-world, cross-sectional study. One-hundred and eleven adolescents aged 12–17 who access the institute of hospitalization and care with scientific character (IRCCS) Mondino Foundation Neuropsychiatric services (Pavia, Italy) between 2017 and 2020 and their parents (*n* = 222) were included. Sociodemographic characteristics of adolescents and their parents were collected. Family functioning was evaluated through the Family Adaptability and Cohesion Evaluation Scale-IV (FACES-IV) and the level of stress through the Perceived Stress Scale (PSS). Twenty adolescents had EOP, 38 had CHR-P, and 59 had no CHR-P. In total, 2.6% of CHR-P adolescents were adopted, 76.3% had separated-divorced parents, and 34.2% of parents had a depressive disorder. Among the FACES-IV sub-scale, maternal rigidity was progressively increased from no-CHR-P to CHR-P to EOP group, with statistical differences between EOP and the other two groups (*p* = 0.01). CHR-P mothers and fathers showed a high level of PSS values, without group difference. Lastly, PSS values correlated positively with the Rigidity, Disengagement, and Chaos scale of FACES-IV and negatively with the Communication scale (*p* < 0.05). Our results suggest that family functioning has a central role and could represent a worthwhile target of intervention for adolescents at CHR-P, leading the way to new preventive approaches.

## Introduction

Psychotic disorders typically onset in adolescence and early adulthood (1), with a mean peak age at onset of 20.5 years (2). Once the disorder onsets, the opportunities to improve their course are limited (3). Therefore, early intervention and preventive approaches [termed “primary indicated prevention” (4–6)] in young people with clinical high risk for psychosis (CHR-P) have the potential to benefit their lives.

Recent studies and umbrella reviews indicate that CHR-P shows more comorbid mental disorders (7, 8), poorer functioning (1, 8–12), worse family relationships (13), higher level of perceived stress (14), and worse long-term outcomes (15) than healthy controls. CHR-P has about a 20% risk of developing psychosis at 2 years (16) but only one-third of them will eventually recover from their initial at-risk symptoms (15). On the contrary, less empirical evidence is available for what concerns effective preventive treatments (7), and it is currently insufficient to favor Cognitive Behavioral Therapy (5, 17). For what concerns family interventions, out of only three psychotherapeutic trials on family dynamics, two showed that an intervention on CHR-P adolescents and their parents improved the attenuated psychotic symptoms over time (7). Specifically, the parental role was identified as a determining factor that shows a negative correlation between mother’s criticism and improvement in the severity of symptoms at 12 months (18). However, these potential preventive effects are not consolidated.

In this regard, interventions that involve parents already represent a focal point for adolescent treatment in other psychiatric disorders (8). In fact, parental couples may incorrectly perceive the family dynamics when their child is affected by severe psychiatric disorders, leading to high levels of perceived stress. Therefore, in this point of view, dysfunctional family functioning and perceived stress could be both a consequence of psychiatric disorder and one of the risk factors that could persevere and increase the severity of symptoms (19). For these reasons, family functioning may represent a worthwhile target of intervention for adolescents at CHR-P (20). However, no comprehensive information about parental pair in CHR-P is available from the present literature, which is instead focused on adolescent perceptions and experiences (21, 22).

From currently knowledge is that both family relationships and social stress could impact the course and outcome of the illness (14). In detail, CHR-P adolescents report high levels of family conflict, childhood maltreatment, and general feelings of social stress when compared to healthy controls (22–24). Moreover, CHR-P adolescents living in less cohesive and supportive family environments show more severe symptoms, more functional impairment, and increased risk of symptom relapse (13, 25). While parents warmth, positive remarks, and involvement predict improvements in symptoms and social functioning (26), perceived stress is often associated with greater baseline symptom severity, progression, and increased likelihood of conversion to psychosis (13, 26, 27).

Overall, if on the one hand, the role of positive family relationships as a protective factor is clear (14), on the other, there is a lack of knowledge, especially about parents’ dynamics and functioning. The present study proposes to address this issue. Our hypothesis is that there may be specific characteristics in family functioning that could be identified as prognostic factors and, consequently, as possible targets for intervention. At the same time, it may also be important to identify the main sources of perceived stress in the parental couple in order to help in targeting future interventions. Specifically, we would like to investigate our hypotheses that compare family functioning and the stress perceived by parents of adolescents with either CHR-P, early onset psychosis (EOP), or other psychiatric disorders. As a secondary aim, it will correlate family functioning with parental perceived stress in order to find critical targets of intervention.

## Materials and Methods

### Design

This cross-sectional study was conducted according to the Reporting of Studies Conducted Using Observational Routinely-Collected Health Data (RECORD) Statement (see Supplementary Table 1) and has received ethical approval from the local ethics committee (P-20170028892). It is a part of the largest research protocol, which is explained in Molteni et al. (28).

### Study Population

All help-seeking adolescents aged 12–17 years and their parents, consecutively admitted to the Child and Adolescent Neuropsychiatric Inpatient and Outpatient Units of institute of hospitalization and care with scientific character (IRCCS) Mondino Foundation (Pavia, Lombardy, Italy) between January 2017 and October 2020, were eligible to be recruited in this study.

Then, we applied the following exclusion criteria for parents: (i) absence of two caregivers for each family; (ii) cognitive and/or psychiatric problems of adolescent’s parents that could compromise the completion of study tests. The parental couples were included even if they were divorced/separated.

Indeed, exclusion criteria for adolescents were: (i) the previous history of any psychotic disorder according to Diagnostic and Statistically Manual of Mental Disorders 5 (DSM-5) (29), (ii) head injuries or any other underlying medical/neurological conditions, (iii) current DSM-5 illicit substance dependence or illicit substance-induced mental disorders, (iv) presence of Brief and Intermittent Psychotic Symptoms (BLIPS), according to Comprehensive Assessment of At Risk Mental State (CAARMS) criteria (30–32), and (v) established Wechsler Intelligence Scale for Children-IV (WISC-IV) (33) or Wechsler Adult Intelligence Scale-IV (WAIS-IV) (34) cognitive impairment (IQ < 70).

Once participants and their parents provided written consents, study enrollment was confirmed.

Participants were then divided into three groups as follows: (i) adolescents with established EOP (EOP hereby), (ii) adolescents meeting CAARMS CHR-P criteria (30) (CHR-P hereby), (iii) adolescents with other DSM-5 psychiatric disorders who did not meet Attenuated Psychotic Syndrome (APS)/EOP criteria (no CHR-P hereby).

### Study Measures

#### Baseline Variables

Upon study entry, CAARMS (35) was carried out for all adolescents, allowing to divide them into the three abovementioned groups. For adolescents in no CHR-P group, other DSM-5 diagnosis than APS/EOP was identified (see Supplementary Methods 1). In addition, we evaluated:

(i) sociodemographic characteristics for both adolescents and parents. The presence of adoptive family or separated parents and Socio-Economic Status (SES) were also included as variables (36);
(ii) family history of any DSM-5 psychiatric disorders evaluated by two independent clinicians, in detail, we assessed by clinical interview the familiarity for psychiatric disorders presented in both parents and grandparents (first and second degrees) of the adolescents enrolled in the study.
(iii) perceived familial functioning and level of stress through self-administered questionnaires given to both parents (see below).

#### Perceived Familial Functioning Variables

We had investigated these aspects through a self-rated questionnaire, Family Adaptability and Cohesion Evaluation Scale-IV (FACES-IV) (37), administered to both parents independently.

In detail, this questionnaire was formulated to provide a qualitative and quantitative evaluation of family functioning. It is composed of different items to evaluate:

(i) Balanced scales: these are termed “balanced” because they are directly proportional to the family’s wellness, with they have a positive correlation. Balanced scales are represented by “cohesion” and “flexibility”;
(ii) Unbalanced scales: on the contrary, “unbalanced” scales represent features of family functioning that are considered extreme (in detail, as extreme areas of flexibility and cohesion). As extreme features, they are considered negative and, consequently, inversely related to family wellness. These are represented by “Disengagement,” “Enmeshment,” “rigidity,” and “Chaos.”
(iii) “Communication” and “satisfaction” scale: they represent the ability of family members to recognize the levels of communication and satisfaction in their own family.
  (iv) Ratio measures between balanced and unbalanced scale, representing a global familial functioning and represented by:
  - “cohesion ratio” = (*cohesion*)/[(*disengagement* + *embeshment*)/2
  - “flexibility ratio” = (*flexibility*)/[(*rigidity* + *cahos*)/2
  - “global ratio” = (*cohesionratio* + *flexibilityratio*)/ 2

All measures have a level of functioning according to FACES-IV score—both balanced and unbalanced scales are divided into three levels of functioning (dysfunctional, intermediate, and functional), communication and satisfaction scale in four levels of functioning (low, intermediate, good, and very good), and ratio measures in two (dysfunctional and functional).

#### Perceived Stress Variables

Perception of stress takes place when a subject realizes that situational demands exceed their resources. In this regard, in our study, we have investigated the level of stress through the self-administered questionnaire Perceived Stress Scale (PSS-10), administered by both parents independently. It was a stress scale composed of 10 items, previously validated (38) and used in other studies (39). Answers are given on a 5-point Likert scale ranging from 1 (never) to 5 (very often), with higher scores reflecting greater perceived stress. A score above 14 is considered to reflect significant perceived stress (40).

### Outcome Measures

The primary outcome was to compare family functioning (using FACES-IV) and level of stress (as measured by PPS-10 scores) in parents of adolescents with CHR-P, EOP, and no CHR-P. The secondary outcome was to correlate family functioning variables (using FACES-IV sub-scale) to the level of stress (PSS-10 scores), considering the whole sample in order to test whether specific familial characteristics could be associated with greater stress.

### Statistical Analysis

Descriptive analyses included median, first, and third quartiles, mean values, and standard deviation (SD), as appropriate for continuous variables, absolute and relative frequencies for categorical variables. Descriptive analyses were complemented by statistical comparisons between the three groups. Bivariate correlation analyses and Kruskal-Wallis were used for numerical variables and chi-square test for categorical variables, complemented by *post-hoc* analyses (Dunn test and Fisher test, respectively, appended supplementary). To reduce the chance of type I error due to multiple testing, Bonferroni correction was applied to all *post-hoc* analyses. More specifically, to test our main hypothesis, 24 (12 FACES-IV scales × 2 parents) comparisons between the three groups were performed using a series of Kruskal-Wallis tests with Bonferroni correction. We expected a 4.8% chance^1^ of observing at least one significant result.

Data were analyzed using R (48); all tests were two-sided, with alpha set at 0.05. All authors have complete access to our database, in which data were collected only after pseudonymization.

## Results

### Study Population

**FIGURE 1:**
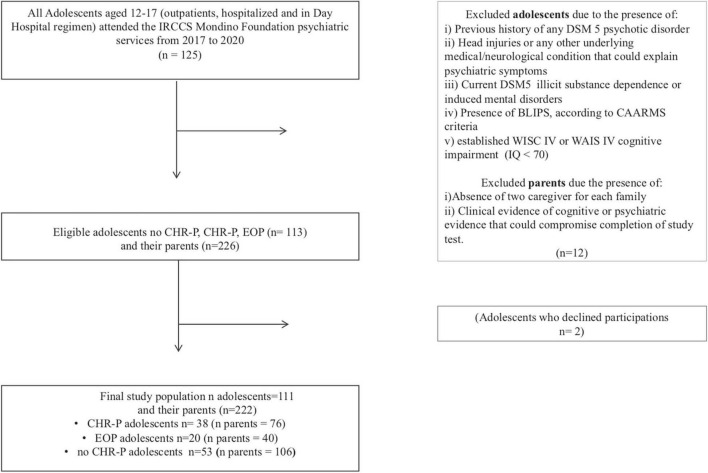
Flow chart of the study population.

The flow chart of the study population is shown in Figure 1. In total, 111 adolescents and their parents (*n* = 222) were included. Among adolescents, 38 were in the CHR-P group, 20 in EOP, and 53 in no CHR-P groups (see Supplementary Table 3 for the specific psychiatric disorders of this subgroup). Across the CHR-P group, all adolescents met APS criteria and none met additionally Genetic Risk and Deterioration Syndrome (GRD) criteria.

### Sociodemographics

The average age of adolescents was 15.1, 65.8% of them were women, and 92.1% were Italians.

Concerning parental information, 2.6% of adolescents were adopted, 76.3% from separated-divorced families. In our sample, there were not same-sex parental couples. The median SES, evaluated through Hollingshead Four Factor Index of SES scale, was 31.5 [interquartile range (IQR) 20.7–40.1]. Both for adolescents’ and families’ sociodemographic characteristics, there were no between-group differences (see Table 1).

**TABLE 1:** Sociodemographics and family history of psychiatric disorders in the total adolescent sample and the clinical high risk state for psychosis (CHR-P), no CHR-P, and early onset psychosis (EOP) subgroups.

Characteristic	Total (*N* = 111)	No CHR-P (*N* = 53)	CHR-P (*N* = 38)	EOP (*N* = 20)	*p*
**Sociodemographics**					
Age, years, median (min, max)	15.0 (12.0, 17.9)	14.9 (12.1, 17.9)	15.1 (14.5, 16.7)	15.6 (12.2, 17.2)	0.495
Sex, female, n (%)	81 (69.2)	44 (74.6)	25 (65.8)	12 (60.0)	0.406
Ethnicity, n (%)					0.418
Italian	103 (88.0)	49 (83.1)	35 (92.1)	19 (95.0)	
Northern African	1 (0.9)	–	1 (2.6)	–	
Albanian	2 (1.7)	2 (3.4)	–	–	
Eastern European	4 (3.4)	3 (5.1)	–	1 (5.0)	
Other	7 (6.0)	5 (8.5)	2 (5.3)	–	
Socio economic status, median (IQR25, 75)	32.3 (22.0,39.5)	33.0 (22.5, 41.0)	31.5 (20.7, 40.1)	30.7 (22–32.1)	0.629
Adopted, n (%)	5 (4.3)	4 (6.8)	1 (2.6)	–	0.359
Separated-divorced family, n (%)	35 (29.9)	36 (61.0)	29 (76.3)	17 (85.0)	0.077
**Family history of any DSM-5 psychiatric disorders, n (%)**					
None	43 (36.8)	19 (32.2)	17 (44.7)	7 (35.0)	0.451
Psychosis	7 (6.0)	2 (3.4)	2 (5.3)	3 (15.0)	0.163
Depression	40 (34.2)	18 (30.5)	13 (34.2)	9 (45.0)	0.498
Anxiety	20 (17.1)	12 (20.3)	5 (13.2)	3 (15.0)	0.633
Substance abuse	9 (7.7)	5 (8.5)	2 (5.3)	2 (10.0)	0.772
Disruptive disorder	3 (2.6)	1 (1.7)	1 (2.6)	1 (5.0)	0.721
Eating disorder	5 (4.3)	4 (6.8)	1 (2.6)	0 (0.0)	0.359
Other^a^	29 (24.8)	13 (22.0)	10 (26.3)	6 (30.0)	0.229

*^a^Includes attention deficit hyperactivity disorders, tics, post-traumatic disorder.*

### Family History of Psychiatric Disorders

Lack of positive family history of any mental disorder was present in 44.7% of CHR-P families; family history of psychosis was traceable in 5.3% of participants and the most frequent DSM-5 diagnosis was of depression disorders (34.2%); there were no between-groups differences (Table 1).

### Perceived Mothers’ Family Functioning

Clinical high risk state for psychosis adolescent’s mothers showed on average intermediate values for both balanced and unbalanced scales and respectively: 45.45 cohesion, 46.89 flexibility, 60.66 Disengagement, 45.68 Enmeshment, 46.68 Rigidity, and 57.63 Chaos. Communication and satisfaction scales were, respectively, mean 33.13 and 31.29. The mean ratio values were 1.11 for the Cohesion Ratio, 1.27 for the Flexibility Ratio, and 1.04 for the Global Ratio (see Table 2).

**TABLE 2:** Results of perceived family functioning and stress in both mothers and fathers independently in the whole sample and in the clinical high risk state for psychosis (CHR-P), no CHR-P, and early onset psychosis (EOP) subgroups.

	Mother					Father				
Characteristic	Total (*N* = 111)	No CHR-P (*N* = 59)	CHR-P (*N* = 38)	EOP (*N* = 20)	*p*	Total (*N* = 111)	No CHR-P (*N* = 59)	CHR-P (*N* = 38)	EOP (*N* = 20)	*p*
**FACES IV** **mean (IQR, 25%, 75%)**										
Cohesion	49.26 (27.5, 70)	51.73 (30, 80)	45.45 (30, 62.5)	49.20 (21.3,77.7)	0.647	50.27 (30, 80)	51.56 (30, 80)	47.24 (25, 72.5)	52.25 (27.5, 80)	0.686
Flexibility	49.68 (25, 70)	52.61 (30, 75)	46.89 (25, 70)	46.30 (21.3, 70)	0.416	50.18 (30, 70)	50.14 (30, 70)	46.82 (30, 62.5)	56.70 (26.2, 85)	0.379
Disengaged	51.89 (30, 75)	48.88 (20, 75)	60.66 (40, 85)	44.10 (6.3, 75)	0.083	49.15 (30, 75)	47.19 (20, 70)	51.63 (30, 80)	50.20 (19.5, 78.8)	0.699
Enmeshed	43.66 (25, 60)	40.78 (25, 60)	45.58 (30, 62.5)	46.60 (26.3, 70)	0.404	48.87 (27.5, 70)	45.44 (25, 70)	53.87 (25, 81.3)	49.50 (31.3, 67.5)	0.372
Rigid	46.38 (20, 70)	40.95 (20, 60)	46.68 (23.75, 70)	61.85 (50, 80)	0.**008**	44.50 (25, 60)	40.90 (20, 60)	45.82 (25, 70)	52.65 (32.5, 73.7)	0.197
Chaos	54.85 (40, 77.5)	52.41 (40, 75)	57.63 (30, 83)	56.75 (40, 80)	0.326	58.80 (35, 83)	56.15 (30, 83)	62.32 (40.7, 83)	59.95 (30, 83)	0.639
Communication	34.40 (29, 40)	35.37 (30, 40)	33.13 (27, 39.5)	33.95 (27.3, 41.8)	0.388	34.40 (29, 40)	35.34 (31, 40)	33.00 (27.7, 38)	34.30 (26.2, 42.7)	0.281
Satisfaction	32.27 (27, 38)	33.24 (28, 39)	31.29 (25, 38)	31.30 (23.8, 36.8)	.478	32.74 (27, 38)	33.97 (30, 39)	30.45 (25, 36.2)	33.45 (26.3, 42.7)	0.**050**
Cohesion ratio	1.40 (0.6, 1.5)	1.44 (0.7, 1.5)	1.11 (0.6, 1.5)	1.82 (0.4, 1.4)	0.107	1.21 (0.6, 1.6)	1.29 (0.7, 1.7)	1.05 (0.6, 1.7)	1.26 (0.8, 1.7)	0.512
Flexibility ratio	1.47 (0.5, 1.8)	1.79 (0.5, 0.2)	.96 (0.4, 1.5)	1.52 (0.6, 1.5)	0.158	1.54 (0,6, 1.5)	1.61 (0.6, 1.7)	1.12 (0.4, 1.3)	2.15 (0.5, 1.4)	0.266
Global ratio	1.44 (0.6, 1.6)	1.62 (0.9, 1.8)	1.04 (0.5, 1.5)	1.67 (0.4, 1.5)	0.178	1.38 (0.6, 1.6)	1.45 (0.5, 1.7)	1.08 (0.6, 1.7)	1.71 (0.5, 1.7)	0.330
PSS	19.84 (14, 25)	19.05 (12, 25)	21.16 (15, 27)	19.65 (14.2, 24.7)	0.266	17.45 (12, 23)	16.42 (11, 21)	18.24 (11.8, 24.2)	19.00 (14, 24.7)	0.192

*Bold values indicate p values with statistic relevance.*

**FIGURE 2:**
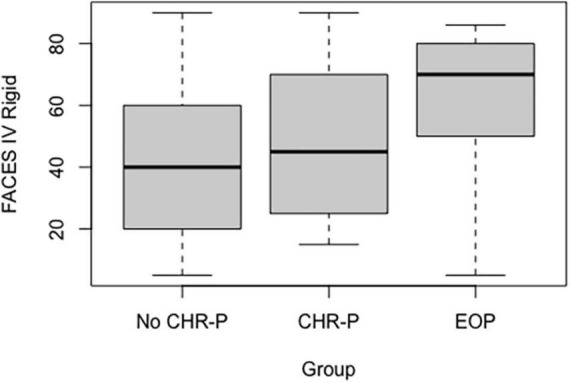
Mothers’ rigidity subscale in the three subgroups.

Regarding group comparison, we found only a significant difference with respect to the rigidity subscale (*p* = 0.008). In detail, mothers of the EOP group were shown to perceive more rigidity than both no CHR-P (*p* = 0.002) and CHR-P groups (*p* = 0.028; see Figure 2). On the contrary, there was not a significant difference between mothers of CHR-P and no CHR-P adolescents (see Supplementary Table 2).

### Perceived Fathers’ Family Functioning

As mothers, also CHR-P adolescent’s fathers showed intermediate mean values for both balanced and unbalanced scales and respectively: 47.23 cohesion, 46.82 flexibility, 51.63 Disengagement, 53.87 Enmeshment, 45.81 Rigidity, and 62.31 Chaos. Communication and satisfaction scales were, respectively, mean 33.00 and 30.44. The mean ratio values were 1.05 for the Cohesion Ratio, 1.12 for the Flexibility Ratio, and 1.08 for the Global Ratio (see Table 2).

**FIGURE 3:**
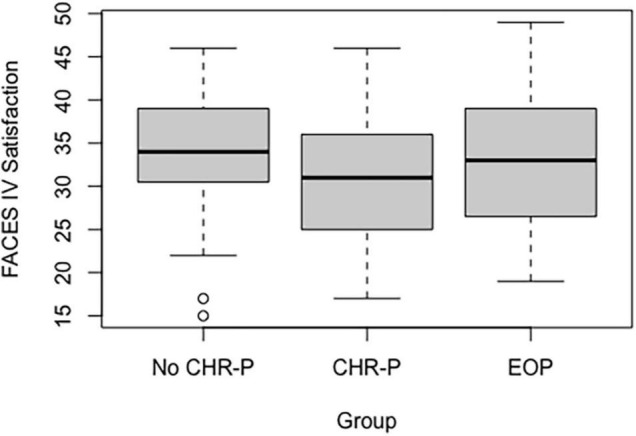
Fathers’ satisfaction subscale in the three subgroups.

Regarding group comparison, we found only one significant difference in the satisfaction subscale (*p* = 0.05), with more perception of satisfaction in fathers of no CHR-P as compared to CHR-P group (*p* = 0.015) (see Figure 3 and Supplementary Table 2).

### Perceived Mothers’ Stress

The mean value of perceived stress of CHR-P adolescents’ mothers was 21.16, showing a high and significant level of perceived stress (see Table 2). There were no between-group differences.

### Perceived Fathers’ Stress

As mothers, also fathers show a high and significant level of perceived stress with a mean value of 18.23 (see Table 2). There were no between-group differences.

### Pearson Correlation Between Family Functioning and Perceived Stress

Among Faces-IV scales and PSS-10 level of stress, we found weak/moderate positive correlations for both mothers and fathers between the level of perceived stress and values of Disengaged (*p* < 0.05 for both mothers and fathers), Rigid (*p* < 0.01 for mothers and *p* < 0.05 for fathers), and Chaos (*p* < 0.01 for both mothers and fathers). In addition, for only mothers, there was a negative correlation between the level of stress and the Communication scale (*p* < 0.05; see Table 3).

**TABLE 3:** Pearson correlation between family adaptability and cohesion evaluation scale-IV (FACES-IV) scales and perceived stress scale (PSS) in the whole sample.

FACES IV	PSS	
	Mother	Father
Cohesion	–0.129	–0.104
Flexibility	–0.052	–0.106
Disengaged	0.216*	0.193*
Enmeshed	0.119	0.020
Rigid	0.307**	0.207*
Chaos	0.384**	0.323**
Communication	−0.192*	–0.292
Satisfaction	–0.248	–0.337
Cohesion ratio	–0.171	–0.285
Flexibility ratio	–0.024	–0.113
Global ratio	–0.111	–0.180

**p < 0.01; *p < 0.05.

See Supplementary Results 1 for Pearson correlation between mothers and fathers in both FACES-IV subscales and PSS values.

## Discussion

Until now, there are no comprehensive studies in the literature that have investigated the role of the parental couple in CHR-P adolescents. In addition, to the authors’ knowledge, the studies with reliable sample sizes did not investigate family functioning (7, 8). This is thus the largest real-world study that investigated the role of the family in CHR-P adolescents.

Firstly, in our sample, about 75% of CHR-P adolescents come from separated-divorced families: it is known that parental couple’s separation could impact adolescents’ functioning and that may be associated with a higher level of emotional distress (41). However, in our study, this percentage does not differ from other psychiatric disorders (both psychotic and others), underlying how family relationships may have a central role in general psychopathology (19).

Secondly, most CHR-P adolescents had no family history of mental disorders (44.7%). In addition, among those who had psychiatric familiarity, depressive disorders were more frequent than psychotic disorders (34.2 and 5.3%, respectively). Our results were consistent with previous studies in the literature (8). In detail, a recent study showed that depressive symptoms were present in one-third of caregivers of CHR-P individuals, triggering criticism and distress (42). In the same way, a caregiver’s depressive symptoms negatively impact family functioning and promote the insurgence of psychiatric disorders (43). Therefore, previous researches (14, 19) suggested that screening for the presence of emotional distress in families of adolescents accessing mental healthcare could be relevant to orient psychoeducational approaches.

The core finding of the study was that both mothers and fathers of CHR adolescents did not show a specific profile on perceived familial functioning. In all FACES-IV subscales (balanced and unbalanced), we found intermediate values: this suggests that, at presentation, there were no clinically disturbed family relationships. At the same time, the presence of intermediate values showed that caregivers were only partially satisfied with their family functioning and communication, with aspects that were potentially improvable. In this view, a psychotherapeutic approach that aims to encourage an improvement in family dynamics has the potential to be a valid therapeutic intervention (13).

In this context, the most interesting finding concerns maternal rigidity, which was progressively increased from no CHR-P to CHR-P to EOP group. This domain was significantly higher in EOP than in the other groups. Rigidity represents the extreme upper end of flexibility in contrast with the lower end represented by disorganization: mothers with a higher expression of rigidity, therefore, responded positively to questions, such as “In our family when rules are broken, there are severe consequences” or “In our family, there is a rule for every possible situation,” indicating that there were rules and norms within the family that could not be violated (37). This finding is in line with previous literature, as family members of an individual with psychosis are more likely to report extreme family difficulties, such as Disengagement, Rigidity, and Chaos: these families are generally more rigid, less structured, less flexible, and more chaotic (44).

A high maternal rigidity could have a double role: on the one side, mothers could have a restraining function for EOP adolescents that are by definition disorganized in their thought and behavior (25). At the same time, an excessive rigidity may represent a trigger for psychotic symptoms or other types of crises. Therefore, this result also underscores its intrinsic ambivalence: the presence of maternal rigidity could be both a factor preceding the onset of symptoms and a consequence of the disease itself. Indeed, as written above, the disorganization typical of the psychotic adolescent could have favored a more restraining and rigid attitude of family members.

Overall, to date, these findings remain largely unexplored, and their influence on adolescents’ symptoms remains unclear. In the light of the present findings, developing an empirical understanding of factors that initiate and maintain adaptive family functioning in the presence of a psychiatric illness becomes an important research goal for the field of early psychosis intervention (44). For instance, one study reports negative correlations between CHR-P symptoms and maternal criticism (18). If a strict correlation between rigidity and criticism has not yet been studied in CHR-P adolescents, it is true that in other psychiatric conditions, such as eating disorders, rigidity, and criticism, sometimes coexist (45). Similarly, to criticism, rigidity could represent a target of family intervention with the aim to fit the flexibility of the parental couple to the psychopathological characteristics of their child (18). Rigidity could be addressed in psychotherapy focused both on parental-couple or in family-system level: here, could be important to understand and mentalize the deeper emotional states of ourselves and the other, such as the family dynamics. Indeed, a deeper understanding of what happens, especially when a clinical psychopathology occurs, with the sharing of the experience of each member of the family could promote the development of a more balanced family style (not excessively rigid or, on the contrary, not excessively disorganized).

Conversely, it is important to highlight that those familial features cannot be considered as pathogenetic factors themselves for psychosis. Indeed, psychosis remains a multifactorial disease whose pathogenesis is known to have different risk factors, such as, biological factors, stress sensitivity, and environment (3). In this point of view, maternal rigidity could be considered as a risk factor in the context of a predisposition: knowing, however, each individual risk and prognostic factor (i.e., family characteristics) could be a starting point for improving early intervention.

Fourthly, parents’ perceived stress was also investigated. Notably, we found high levels of perceived stress in the CHR-P group for both mothers and fathers, with no difference between EOP and no CHR-P group. The absence of differences between the three groups highlighted the importance of this factor in CHR-P families: it could be a negative factor, such as for adolescents suffering from other psychiatric conditions (both EOP or with other psychiatric disorders) (22, 46). However, as for maternal rigidity, there is a double role of this finding. Indeed, perceived stress may be one of the factors that promote the disease, but at the same time also a consequence of the disease itself (46).

Lastly, correlations between family functioning and perceived stress showed interesting associations: as the imbalance in FACES-IV scales increased, also did the value of perceived stress. Notably, perceived stress increased together with values of Disengagement, Rigidity, and Chaos, which are the three negative domains of the FACES-IV. Disengagement represents distance and lack of involvement within the family, while Rigidity and Chaos are the opposite extremes of good family flexibility (from the most rigid rules to a total lack) (37). The fact that these findings were replicated in mothers and fathers strengthens their validity. Conversely, good communication between family members seems to be important in decreasing perceived stress. In fact, mothers in our sample showed a decrease in perceived stress when family communication was more functional. Overall, these results support the main ones, underlining how a family intervention could be useful. In detail, good cohesion and flexibility may represent a target of family intervention as already shown in other psychiatric disorders, such as eating disorders (45). Although to date, there are no guidelines in the treatment of CHR-P, family therapy could be a good target of intervention. Fostering communication could be important both within the parental couple (with a parental-focused therapy) and with the patients (with a therapy focused on all family members). In this point of view, in the family and parental nucleus, it is of fundamental importance to work on the mentalization and understanding of one’s own and other’s emotions, on the ability to listen and share, even negative experiences, such as the disease itself (45).

The main limitation of the current study is represented by the absence of adolescents’ perceived point of view on perceived familial functioning and stress. However, we will fill this gap in future research that will include all points of view in the family. Another limitation is that since we included adolescents recruited at a third-level center, the CHR-P patients may represent a more severe part of the high-risk spectrum (47).

## Conclusion

Our findings support how family dynamics could be a source of perceived stress and highlight how specific features, such as rigidity, could represent potential directions for family intervention.

## Data Availability Statement

The raw data supporting the conclusions of this article will be made available by the authors, without undue reservation.

## Ethics Statement

This study received ethical approval from the local ethics committee (P-20170028892). Written informed consent to participate in this study was provided by the participants’ legal guardian/next of kin.

## Author Contributions

RB, MM, and MI conceived and designed the study and participated in the acquisition of data. EC analyzed the data. MI drafted the manuscript. PF-P and SD revised the manuscript. All authors have read and approved the final manuscript.

## Conflict of Interest

The authors declare that the research was conducted in the absence of any commercial or financial relationships that could be construed as a potential conflict of interest.

## Publisher’s Note

All claims expressed in this article are solely those of the authors and do not necessarily represent those of their affiliated organizations, or those of the publisher, the editors and the reviewers. Any product that may be evaluated in this article, or claim that may be made by its manufacturer, is not guaranteed or endorsed by the publisher.

## Footnotes

1 To calculate this percentage, we performed the following formula: *p* (at least one significant result) = 1 – *p* (no significant results) = 1 − (1 – α/n)^24^.

## Abbreviations

CHR-P: Clinical High Risk state for Psychosis
DMS-5: Diagnostic and Statistically Manual of mental disorders 5
WISC IV: Wechsler Intelligence Scale for Children IV
WAIS: Wechsler Adult Intelligence Scale
BLIPS: Brief and Intermittent Psychotic Symptoms
CAARMS: Comprehensive Assessment of At Risk Mental State
EOP: Early Onset Psychosis
FACES-IV: Family Adaptability and Cohesion Evaluation Scale-IV
PSS-10: Perceived Stress Scale.
