# Corrigendum: Perceived Family Functioning Profile in Adolescents at Clinical High Risk for Psychosis: Rigidity as a Possible Preventive Target

**DOI:** 10.3389/fpsyt.2022.946506

**Published:** 2022-06-23

**Authors:** Melanie Iorio, Erica Casini, Stefano Damiani, Paolo Fusar-Poli, Renato Borgatti, Martina Maria Mensi, Clinical High Risk State for Psychosis Research Group

**Affiliations:** ^1^Department of Brain and Behavioral Sciences, University of Pavia, Pavia, Italy; ^2^Child Neurology and Psychiatry Unit, Institute of Hospitalization and Care With Scientific Character (IRCCS) Mondino Foundation, Pavia, Italy; ^3^Early Psychosis: Interventions and Clinical-Detection (EPIC) Lab, Department of Psychosis Studies, Institute of Psychiatry, Psychology and Neuroscience, King's College London, London, United Kingdom; ^4^OASIS Service, South London and Maudsley NHS Foundation Trust, London, United Kingdom

**Keywords:** family functioning, psychosis, schizophrenia, risk, prevention, adolescence, CHR-P, perceived stress (PS)

In the published article, there was an error regarding the affiliation**s** for author “Paolo Fusar-Poli,” As well as having affiliations number 3 and 4, he should also have affiliation number 1 “Department of Brain and Behavioral Sciences, University of Pavia, Pavia, Italy.”

The authors apologize for this error and state that this does not change the scientific conclusions of the article in any way. The original article has been updated.

## Publisher's Note

All claims expressed in this article are solely those of the authors and do not necessarily represent those of their affiliated organizations, or those of the publisher, the editors and the reviewers. Any product that may be evaluated in this article, or claim that may be made by its manufacturer, is not guaranteed or endorsed by the publisher.

