# Supplementary material for: Perceived Family Functioning Profile in Adolescents at Clinical High Risk for Psychosis: Rigidity as a Possible Preventive Target
Source: Front Psychiatry. 2022 Apr 15;13:861201. doi: 10.3389/fpsyt.2022.861201 (PMC9051044; doi:10.3389/fpsyt.2022.861201)
Supplement: Supplementary file 1 [file Data_Sheet_1.pdf]

SUPPLEMENTARY MATERIAL

Index

Table S1. The RECORD statement – checklist of items, extended from the STROBE statement, that should be reported in observational studies using routinely collected health data. 2

Table S2. Post-Hoc adjusted p-value for perceived family functioning and level of stress 7

Table S3. Specific psychiatric disorders in no CHR-P group...8

Methods S1 9

Results S1...10

Table S1. The RECORD statement – checklist of items, extended from the STROBE statement, that should be reported in observational studies using routinely collected health data.

	Item No.	STROBE items	Location in manuscript where items are reported	RECORD items	Location in manuscript where items are reported
**Title and abstract**					
	1	(a) Indicate the study's design with a commonly used term in the title or the abstract (b) Provide in the abstract an informative and balanced summary of what was done and what was found	Page 1	RECORD 1.1: The type of data used should be specified in the title or abstract. When possible, the name of the databases used should be included.<br><br>RECORD 1.2: If applicable, the geographic region and timeframe within which the study took place should be reported in the title or abstract.<br><br>RECORD 1.3: If linkage between databases was conducted for the study, this should be clearly stated in the title or abstract.	
**Introduction**					
Background rationale	2	Explain the scientific background and rationale for the investigation being reported	Page 2		
Objectives	3	State specific objectives, including any prespecified hypotheses	Page 2		
**Methods**					
Study Design	4	Present key elements of study design early in the paper	Pages 3-5		
Setting	5	Describe the setting, locations, and relevant dates, including periods of recruitment, exposure, follow-up, and data collection	Pages 3-5		

Participants	6	*(a) Cohort study* - Give the eligibility criteria, and the sources and methods of selection of participants. Describe methods of follow-up<br>*Case-control study* - Give the eligibility criteria, and the sources and methods of case ascertainment and control selection. Give the rationale for the choice of cases and controls<br>*Cross-sectional study* - Give the eligibility criteria, and the sources and methods of selection of participants<br><br>*(b) Cohort study* - For matched studies, give matching criteria and number of exposed and unexposed<br>*Case-control study* - For matched studies, give matching criteria and the number of controls per case	Pages 3-5	RECORD 6.1: The methods of study population selection (such as codes or algorithms used to identify subjects) should be listed in detail. If this is not possible, an explanation should be provided.<br><br>RECORD 6.2: Any validation studies of the codes or algorithms used to select the population should be referenced. If validation was conducted for this study and not published elsewhere, detailed methods and results should be provided.<br><br>RECORD 6.3: If the study involved linkage of databases, consider use of a flow diagram or other graphical display to demonstrate the data linkage process, including the number of individuals with linked data at each stage.	
Variables	7	Clearly define all outcomes, exposures, predictors, potential confounders, and effect modifiers. Give diagnostic criteria, if applicable.	Pages 3-5	RECORD 7.1: A complete list of codes and algorithms used to classify exposures, outcomes, confounders, and effect modifiers should be provided. If these cannot be reported, an explanation should be provided.	
Data sources/ measurement	8	For each variable of interest, give sources of data and details of methods of assessment (measurement). Describe comparability of assessment methods if there is more than one group	Pages 3-5		
Bias	9	Describe any efforts to address potential sources of bias	Pages 3-5		
Study size	10	Explain how the study size was arrived at	Pages 3-5	It is a clinical register-based cross-selectional study concerned all adolescents attended the IRCCS Mondino Foundation psychiatric services from 2017-2019 (see figure 1 Flow chart of the study population)	
Quantitative variables	11	Explain how quantitative variables were handled in the analyses. If applicable, describe which groupings were chosen, and why	Pages 3-5		

Statistical methods	12	(a) Describe all statistical methods, including those used to control for confounding<br>(b) Describe any methods used to examine subgroups and interactions<br>(c) Explain how missing data were addressed<br>(d) *Cohort study* - If applicable, explain how loss to follow-up was addressed<br>*Case-control study* - If applicable, explain how matching of cases and controls was addressed<br>*Cross-sectional study* - If applicable, describe analytical methods taking account of sampling strategy<br>(e) Describe any sensitivity analyses	Pages 3-5		
Data access and cleaning methods		..	Pages 3-5	RECORD 12.1: Authors should describe the extent to which the investigators had access to the database population used to create the study population.<br><br>RECORD 12.2: Authors should provide information on the data cleaning methods used in the study.	
Linkage		..	Pages 3-5	RECORD 12.3: State whether the study included person-level, institutional-level, or other data linkage across two or more databases. The methods of linkage and methods of linkage quality evaluation should be provided.	
**Results**					
Participants	13	(a) Report the numbers of individuals at each stage of the study (*e.g.*, numbers potentially eligible, examined for eligibility, confirmed eligible, included in the study, completing follow-up, and analysed)<br>(b) Give reasons for non-participation at each stage.<br>(c) Consider use of a flow diagram	Pages 5-7	RECORD 13.1: Describe in detail the selection of the persons included in the study (*i.e.,* study population selection) including filtering based on data quality, data availability and linkage. The selection of included persons can be described in the text and/or by means of the study flow diagram.	

Descriptive data	14	(a) Give characteristics of study participants (*e.g.*, demographic, clinical, social) and information on exposures and potential confounders<br>(b) Indicate the number of participants with missing data for each variable of interest<br>(c) *Cohort study* - summarise follow-up time (*e.g.*, average and total amount)	Pages 5-7		
Outcome data	15	*Cohort study* - Report numbers of outcome events or summary measures over time<br>*Case-control study* - Report numbers in each exposure category, or summary measures of exposure<br>*Cross-sectional study* - Report numbers of outcome events or summary measures	Pages 5-7		
Main results	16	(a) Give unadjusted estimates and, if applicable, confounder-adjusted estimates and their precision (e.g., 95% confidence interval). Make clear which confounders were adjusted for and why they were included<br>(b) Report category boundaries when continuous variables were categorized<br>(c) If relevant, consider translating estimates of relative risk into absolute risk for a meaningful time period	Pages 5-7		
Other analyses	17	Report other analyses done—e.g., analyses of subgroups and interactions, and sensitivity analyses	Pages 5-7		
**Discussion**					
Key results	18	Summarise key results with reference to study objectives	Pages 7-9		
Limitations	19	Discuss limitations of the study, taking into account sources of potential bias or imprecision. Discuss both direction and magnitude of any potential bias	Pages 7-9	RECORD 19.1: Discuss the implications of using data that were not created or collected to answer the specific research question(s). Include discussion of misclassification bias, unmeasured confounding, missing data, and changing eligibility over time, as they pertain to the study being reported.	

Interpretation	20	Give a cautious overall interpretation of results considering objectives, limitations, multiplicity of analyses, results from similar studies, and other relevant evidence	Pages 7-9		
Generalisability	21	Discuss the generalisability (external validity) of the study results	Pages 7-9		
**Other Information**					
Funding	22	Give the source of funding and the role of the funders for the present study and, if applicable, for the original study on which the present article is based	Page 9		
Accessibility of protocol, raw data, and programming code		..		RECORD 22.1: Authors should provide information on how to access any supplemental information such as the study protocol, raw data, or programming code.	

Table S2. Post-Hoc adjusted p-value for perceived family functioning and level of stress

	Post-hoc adjusted p-values					
	Mothers			Fathers		
**Characteristic**	**No-CHR vs CHR**	**No-CHR vs EOP**	**CHR vs EOP**	**No-CHR vs CHR**	**No-CHR vs EOP**	**CHR vs EOP**
**FACES IV**						
Cohesion	-	-	-	-	-	-
Flexibility	-	-	-	-	-	-
Disengaged,	-	-	-	-	-	-
Enmeshed	-	-	-	-	-	-
Rigid	0.340	0.002	0.028	-	-	-
Chaos	-	-	-	-	-	-
Communication	-	-	-	-	-	-
Satisfaction,	-	-	-	.015	.609	.176
Cohesion Ratio	-	-	-	-	-	-
Flexibility Ratio	-	-	-	-	-	-
Global Ratio, mean (SD)	-	-	-	-	-	-
**PSS**	-	-	-	-	-	-

Table S3. Specific psychiatric disorders in no CHR-P group

**Specific psychiatric disorders**	**No CHR-P (N=50)**
Depressive disorders	
Major depressive disorder	7(11.9)
Persistent depressive disorder	1(1.7)
Other no-specified depressive disorder	13(22)
Anxiety disorders	
Social anxiety disorder	1(1.7)
Generalized anxiety disorder	2(3.4)
Social phobia	1(.7)
Panic disorder	1(.7)
Other no-specified anxiety disorder	14(23.7)
Bipolar disorder	
Bipolar disorder type 1	1(1.7)
Cyclothymic disorder	1(1.7)
Obsessive-compulsive disorders	
Other no-specified obsessive-compulsive disorder	2(3.4)
Traumatic events related disorders	
Adaptation disorder	2(3.4)
Somatic disorders	
Other no-specified somatic disorder	4(6.8)
Eating disorders	
Anorexia nervosa	3(5.1)
Bulimia	1(1.7)
Other no-specified eating disorder	7(11.9)
Conduct disorders	
Oppositional defiant disorder	1(1.7)
Conduct disorder	1(1.7)
Other no-specified conduct disorder	2(3.4)
Personality disorders	
Under construction	7(11.9)
Borderline	2(3.4)
Narcissistic	1(1.7)
Substance use disorders	1(1.7)
Neurodevelopmental disorders	
Communication disorder	1(1.7)
Autism	2(3.4)
ADHD	1(1.7)
TIC disorder	1(1.7)
Other disorders	19(32.2)

Legend: * in partial remission

Methods S1

As described in the main text, to measure DSM-5 diagnoses for adolescents in no CHR-P group we carried out a diagnostic assessment encompassing self-rated questionnaires administered independently to both parents (Child Behavior Checklist) and adolescents (Youth Self Report) (1) and clinician-rated semi-structured interviews. The latter included the Kiddie-Schedule for Affective Disorder and Schizophrenia Present and Lifetime 5 and Structured Clinical Interview 5 (2)

Results S1

In all three sub-groups we found a positive correlation between mothers and fathers both for FACES IV subscale and PSS values. In detail we found a more statistic relevance in parents of no CHR-P (in cohesion, flexibility, disengaged, enmbeshed, rigidity, satisfaction, cohesion ratio, flexibility and global ratio, PSS value) and of EOP group (in flexibility, disengaged, enbeshed, rigidity, chaos, cominunication, satisfaction, global ratio). Instead in CHR-P group there were a positive correlation statistical relevant only for communication and satisfaction subscales. See table below for all Pearson Correlations.

Table S4. Pearson's correlation between parents on FACES subscales and PSS for each group

	**No CHR-P (n parents= 118)**		**CHR-P (n parents= 76)**		**EOP (n parents=40)**	
	r	*p*	*r*	*p*	*r*	*p*
FACES IV						
Cohesion	**.43**	**<.001**	.28	.083	.29	.214
Flexibility	**.44**	**<.001**	.10	.524	.64	**<.01**
Disengaged	**.36**	**<.01**	.27	.099	.79	**<.001**
Enmeshed	**.45**	**<.01**	.22	.176	.50	**.023**
Rigidity	**.31**	**.020**	.24	.137	.67	**<.01**
Chaos	.24	.068	.40	.014	.67	**<.01**
Communication	.25	.051	.51	**<.001**	.57	**<.01**
Satisfaction	**.40**	**<.01**	.67	**<.001**	.77	**<.001**
Cohesion Ratio	**.30**	**.020**	.20	.237	.42	.067
Flexibility Ratio	**.27**	**.040**	26	.114	27	.248
Global Ratio	**.40**	**<.01**	.23	.155	.97	**<.001**
PSS	**.40**	**<.01**	.01	.957	.01	.957

Legend: in bold Person correlation with p values statical relevant (p <0.05)
